# The Ecology-Culture Dataset: A new resource for investigating cultural variation

**DOI:** 10.1038/s41597-022-01738-z

**Published:** 2022-10-11

**Authors:** Alexandra S. Wormley, Jung Yul Kwon, Michael Barlev, Michael E. W. Varnum

**Affiliations:** Department of Psychology, Arizona State University, Tempe, USA

**Keywords:** Human behaviour, Human behaviour

## Abstract

Scholars interested in cultural diversity have long suggested that similarities and differences across human populations might be understood, at least in part, as stemming from differences in the social and physical ecologies individuals inhabit. Here, we describe the EcoCultural Dataset (ECD), the most comprehensive compilation to date of country-level ecological and cultural variables around the globe. ECD covers 220 countries, 9 ecological variables operationalized by 11 statistical metrics (including measures of variability and predictability), and 72 cultural variables (including values, personality traits, fundamental social motives, subjective well-being, tightness-looseness, indices of corruption, social capital, and gender inequality). This rich dataset can be used to identify novel relationships between ecological and cultural variables, to assess the overall relationship between ecology and culture, to explore the consequences of interactions between different ecological variables, and to construct new indices of cultural distance.

## Abstract (summary)

**Table:** 

Measurement(s)	culture • ecology
Technology Type(s)	archival data

## Background & Summary

Cataloguing and explaining human cultural diversity has been a core question for many branches of the social sciences. Many evolutionary social scientists and behavioral ecologists posit that the patterns of cultural diversity observed around the globe are due, at least in part, to the physical and social ecologies individuals inhabit^1–3^. For example, in societies with a high pathogen prevalence, cultures tend to be more collectivistic—this may be a strategy for dealing with high rates of disease through behaviors that protect the individual and the group from the threat of outside pathogens^4,5^; higher levels of population density have been linked to lower rates of fertility^6,7^, which may reflect an adaptive shift toward slower life history strategies in the face of stiffer social competition; and societies that are afflicted with high extrinsic mortality threats tend to have stronger social norms, which may increase the likelihood of survival in such conditions^8^.

Although investigations of the interplay between ecology and culture have been fruitful, most have focused on links between a single ecological variable (e.g., pathogen prevalence, population density, resources levels) and a single cultural outcome or small set of such outcomes (e.g., individualism-collectivism, tightness-looseness, the Big Five personality traits). Additionally, with some exceptions^7,9,10^, such investigations have focused on levels of those ecological variables at a single time point.

Here, we present the EcoCultural Dataset (ECD)^11^, which aims to address these limitations and spur future discoveries. The ECD is a compilation of data from 220 countries on nine ecological variables and 72 cultural variables that are likely to be of broad interest to social scientists. The country-level data in the ECD complements other comprehensive data sources such as D-PLACE, which focuses on small-scale societies^12^. The ECD includes time series data on ecological variables and 11 statistical metrics for each, designed to index properties such as historical averages, variability across time, predictability, and extreme perturbations.

## Methods

### Variable selection

Data were collected on the level of countries. In constructing the ECD^11^, we selected only variables that contained data from a minimum of twenty countries.

#### Ecological variables

Although conceptualizations of ecology vary across disciplines^13^, they generally share an emphasis on the relationships between organisms and their external environment^14^ Our conceptualization of ecology is largely grounded in behavioral ecology^15^ and extends beyond the physical environment to include key features of the social environment as well which have been linked to adaptive responses across species, such as population density and resource availability^2^. This conceptualization of ecological variables encompasses environmental conditions that have direct implications for an individual’s survival and reproduction, which includes not only aspects of the natural environment related to climate, but also factors like the availability and distribution of resources key to biological fitness.

We required that all ecological variables contain at least 20 time points for some number of countries to conduct a sufficiently powered time series analysis using the ARIMA model. We gathered data on nine different ecological variables: rainfall, temperature, GDP per capita, income inequality, external mortality, life expectancy, disease threat, population density, and unemployment rates (Table 1). Although these variables capture a wide range of features of the physical and social environment that may have consequences for human cultural variation, we do not argue that this is an exhaustive set of variables which could be considered ecological.

**Table 1:** Ecological variables included in the Ecology and Culture Dataset (ECD).

Variable	Description	Source	Year	*N* Societies
Rainfall	Average rainfall per year (mm)	Climate Change Knowledge Portal^35^	1901–2016	195
Temperature	Average temperature per year (°C)	Climate Change Knowledge Portal^35^	1901–2016	195
GDP	GDP per capita (in USD)	The World Bank^36^	1960–2019	198
Gini	Operationalization of income inequality	Standardized World Income Inequality Database^37^	1960–2017	126
Mortality	Rates of mortality per 100,000 people from external causes (i.e. accidents, interpersonal violence)	World Health Organization^38^	1979–2016	96
Life Expectancy	Life expectancy at birth, total (years)	The World Bank^39^	1960–2018	196
Disease Threat	Percentage of total deaths in the population due to HIV/AIDS, respiratory infection, enteric infections, and other communicable infections	Global Burden of Disease Collaborative Network, 2020^40^	1990–2019	204
Population Density	People per km²	Rotella *et al*.^7^	1950–2019	217
Unemployment	Total % of the labor force that is unemployed	The World Bank^41^	1960–2019	101

A similar table is presented in Wormley *et al*.^21^.

#### Cultural variables

Cultural variables were identified through reviews of the psychological literature and by crowdsourcing on social media and Listservs from the psychology community. To be included in the ECD, cultural variables needed (a) to be indices (not single-item measures) and (b) to contain data for at least 20 countries. In total, this search yielded 72 cultural variables including values, personality traits, motivations, social norms, subjective well-being, innovation, and government functioning (see data for full list).

#### Data usage and permissions

Ecological variables were collected from the World Bank, World Health Organization, Institute for Health Metrics and Evaluation, and scholarly publications. These sources all permit the use of their data under the CC Attribution 4.0 International License. Full sources are listed in Table 1.

Cultural variables were collected from academic publications or publications by NGOs, intergovernmental organizations, or other public facing data published online, all of which is permitted for use with attribution. The sources of all cultural variables used in the ECD can be found within the datafiles on OSF (https://osf.io/r9msf/).

### Metric calculation

In addition to raw ecological and cultural data, the ECD^11^ includes 11 statistical metrics for each ecological variable (on the level of countries) to allow researchers to explore the relationship between time-variant features of ecological conditions and present-day cultural variation: current levels, mean across time, extreme perturbations (maximum, minimum), as well as indicators of trends (standardized linear regression coefficient), variability across time (standard deviation, range, percentage of outliers), and predictability (Mean Absolute Percent Error, Mean Absolute Standard Error, first-order autocorrelation). We calculate these metrics for years ranging from 1950 to 2020.

1. Current Level: Datapoint for the year of publication for the corresponding cultural variable, or the most recent available data point.
2. Mean Level: Arithmetic average of all available datapoints.
3. Standard Deviation (*Variability*): Average deviation from the mean.
4. Range (*Variability*): Difference between the maximum and minimum values.
5. Maximum Value (*Extreme Perturbation*): Highest datapoint in the dataset.
6. Minimum Value (*Extreme Perturbation*): Lowest datapoint in the dataset.
7. Mean Absolute Percentage Error (MAPE; *Predictability*): Derived from auto.ARIMA and a train-test procedure (see below), an accuracy measure defined as the absolute, average percent deviation between the actual value in a time series and the forecasted value from an ARIMA model. Higher percentages indicate more error, or less predictability.
8. Mean Absolute Scaled Error (MASE; *Predictability*): Derived from auto.ARIMA and a train-test procedure (see below), an accuracy measure defined as the ratio of errors made by an ARIMA model relative to a naïve forecast. Higher values indicate more error, or less predictability.
9. First-Order Autocorrelation (*Predictability*): Correlation between successive residuals in a time series, with greater values indicating a high degree of relatedness between time, *t*_*n*_, and the successive time point, *t*_*n+1*_. Lower values indicate less similarity between time points, or less predictability.
10. Percent of Outliers (*Extreme Perturbation*): Percent of datapoints in a time series that deviate more than 2.5 standard deviations from the mean.
11. Standardized Beta Coefficient (β): Measure of the linear relationship between time and the ecological variable, derived from a linear regression analysis where the data have been standardized such that the standard deviation of the ecological and cultural variables equal 1 and their respective means equal 0. Lower values indicate a less gradual linear increase (or decrease, for negative values) over time.

Thus, we calculated 99 different estimates of ecology *for each country* for which data was available—eleven metrics for each of the nine ecological variables (current levels of rainfall in Ukraine, mean rainfall in Ukraine, etc.).

### auto.ARIMA

MAPE and MASE values were calculated using the auto.ARIMA function from the *forecast* package in R^16^, a machine learning algorithm that fits models with various AutoRegressive Integrated Moving Average (ARIMA) parameters to a time series dataset and selects the optimal model based on fit. We used a two-step train-test procedure. In the first step, auto.ARIMA was used to fit a model based on 80% of datapoints. In the second step, that model was fit to the held-out portion of 20% of datapoints. We gathered three measures of predictability from these analyses: MAPE and MASE (where predictability for a given ecological variable was operationalized as the amount of error), and first-order autocorrelation.

## Data Records

All data is available on the Open Science Forum at https://osf.io/r9msf/^11^.

1. **ECD Codebook:** Contains the meta-data for all ecological and cultural variables contained within the dataset, including source, whether the variable is a part of a larger taxonomy (e.g., Big Five, Moral Foundations), sample size when available, and variable type (e.g., continuous/discrete, raw/transformed).
2. **ECD Data:** Contains raw country-level data for nine ecological variables, 72 cultural variables, operationalizations (calculated based on all the time series data including and proceeding that year), and geographic meta-data (latitude, longitude, World Health Organization world region). Because this file presents time series data, it is organized in the “long-format,” such that every row represents a country and year, with columns representing specific ecological variables. If calculating correlations between an ecological variable and a cultural variable using time series data, it is important to truncate the time series of the ecological variable to *before* the data collection of the cultural variable. For example, if correlating mean rainfall and Agreeableness, calculate a country’s mean rainfall from the first available date to 2007, which is the year of publication for the Agreeableness variable, not 2019, the last available year of data for rainfall.

## Technical Validation

The specific measures used to calculate the variables are available in their original source material. The scales for the ecological variables are available in Table 1. Missing data are entered as “NA”. In the case of the “Codebook”, NA under “Sample Size” indicates that there was no sampling (in the case of variables like “rainfall” or “Human Development Index”) or that the exact sample size is not provided (in the case of the Hofstede variables).

It is worth noting that spatial and temporal autocorrelation are issues that researchers may encounter when using this dataset. Countries in geographical proximity should not be considered as independent datapoints, due to high probability of shared ancestral history and horizontal cultural transmission^17,18^. Further, ecological data from two consecutive years are likely highly correlated given that it is rare for ecological conditions to drastically change from year to year and our ecological data is often averaged or collected at a single time point within a year. Spatial autocorrelation can be addressed in many ways, including by conducting analyses within world regions (to control for shared cultural history) or by using statistical approaches such as autocovariate models^19^. Temporal autocorrelation can be addressed through various methods for detrending time series data such as differencing or residualizing out linear trends and autoregressive components^10,20^.

## Usage Notes

ECD can be used to explore theoretically and methodologically important questions about the ecology and human cultural universality and diversity. By analyzing these data on their own or in concert with other sources of data, researchers can explore questions including:

- How much of human cultural variation around the globe is explained by ecology?^21^
- How might *interactions* between different ecological variables and/or their statistical metrics be linked to both specific cultural variables and patterns of cultural variation in general?
- Are historical ecological conditions or current ecological conditions more closely related to cultural variation?^21^
- Do different ecological metrics (such as predictability versus current levels) have qualitatively different linkages to cultural diversity?^21^
- How might interactions between different metrics of the same ecological variable be linked to cultural variation?
- How might interactions between certain ecological variables (or their statistical metrics) and cultural variables be linked to cultural variation?
- Do societies cluster in a similar or different fashion based on different cultural variables?
- How might ecological similarity between home and host society predict relative ease or difficulty of acculturation?

### Illustrative exploratory analysis

To illustrate one promising way in which these data can be used, we explore—visually, using dendrograms—how countries cluster based on cultural and ecological variables. Dendrograms are depictions of hierarchical clustering, a statistical method for grouping similar observations within a dataset across multiple hierarchically nested levels. The degree of similarity between two dendrograms can be assessed using Baker’s gamma—a rank correlation coefficient with corrections for non-independence of observations^22^, or rather, whether or not countries cluster at the same hierarchical level across two dendrograms. As with a Pearson coefficient, Baker’s gamma values range from −1 (highly dissimilar) to 1 (highly similar). We note that although dendrograms have been used in prior cross-cultural work to explore clustering of countries^23–25^, this approach is primarily used in an exploratory fashion, as is the case in the present work. Thus the present analyses maybe better thought of as a jumping off point for further investigation rather than definitive findings.

**Fig. 1:**
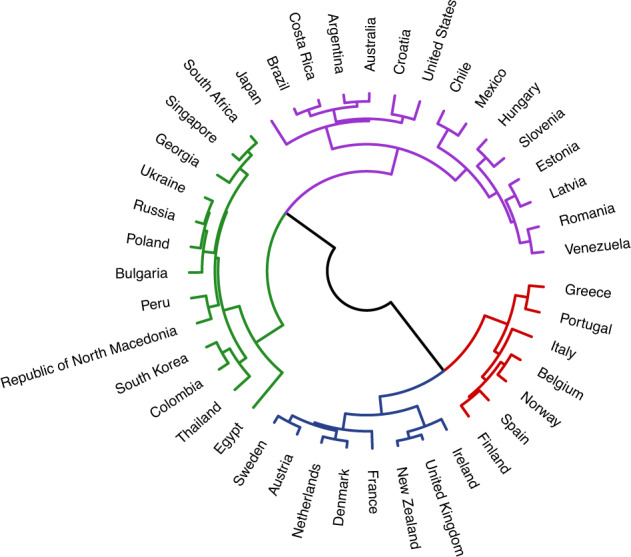
Dendrogram representing Schwartz’ value orientations for 43 countries. The dendrogram branches diverge at different heights. Smaller heights between links indicate greater similarity (e.g., Sweden and Austria). Greater heights between links indicate lower similarity (e.g., Croatia and United States). Colors represent clusters within the data (fixed to *k* = 4).

**Fig. 2:**
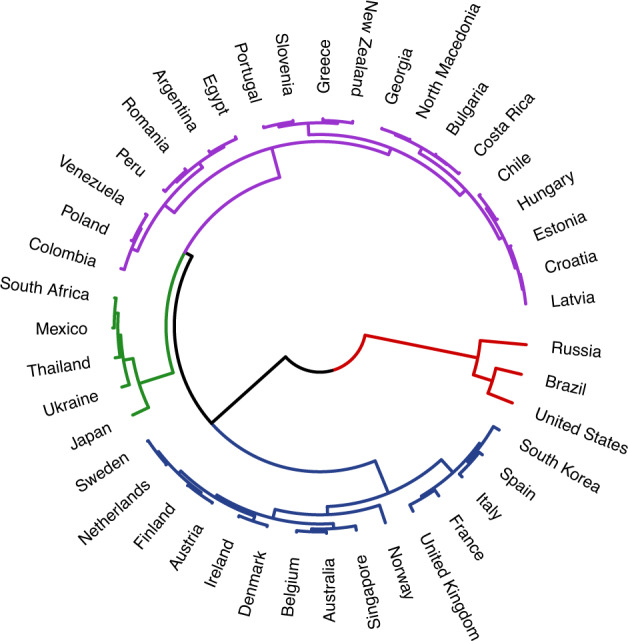
Dendrogram representing current levels of ecology across all 9 ecological variables for 43 countries. The dendrogram branches diverge at different heights. Smaller heights in between links indicate greater similarity (e.g., Slovenia and Portugal). Greater heights between links indicate lower similarity (e.g., Brazil and United States). Colors represent clusters within the data (fixed to *k* = 4).

**Fig. 3:**
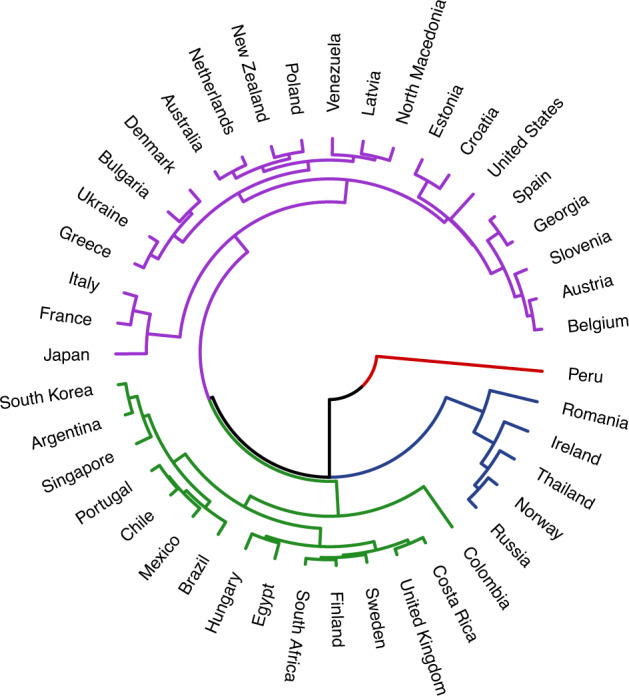
Dendrogram representing one operationalization of ecological predictability (Mean Absolute Percentage Error) for 43 countries. Branches of the dendrogram diverge at various heights. Smaller heights in between links indicate greater similarity (ex. Costa Rica and the United Kingdom). Greater heights between links indicates greater dissimilarity (ex. Estonia and Croatia). The colors represent clusters within the data (fixed to *k* = 4).

**Fig. 4:**
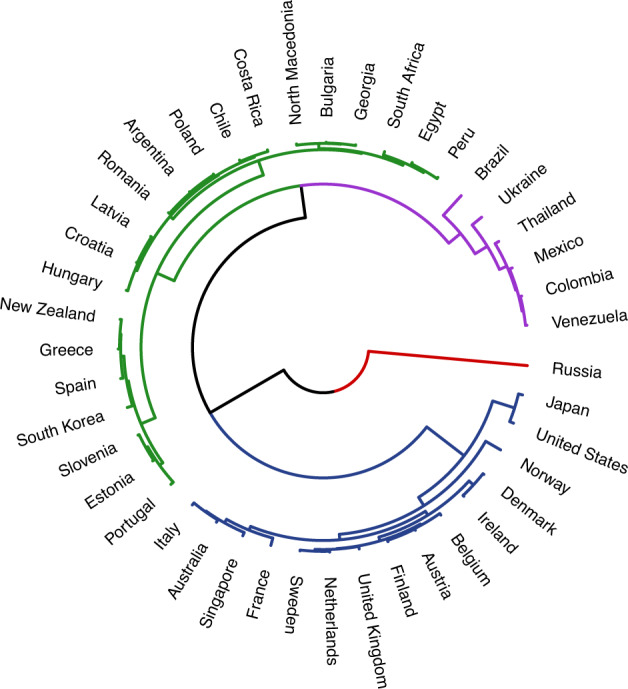
Dendrogram representing one operationalization of ecological variability (standard deviation) for 43 countries. Branches of the dendrogram diverge at various heights. Smaller heights in between links indicate greater similarity (ex. Estonia and Slovenia). Greater heights between links indicates greater dissimilarity (ex. Ukraine and Brazil). The colors represent clusters within the data (fixed to *k* = 4).

We began by building a dendrogram using Schwartz’ cultural values: harmony, embeddedness, hierarchy, mastery, affective autonomy, intellectual autonomy, and egalitarianism^26^ (Fig. 1). Next, we built a series of dendrograms representing the eleven different metrics (in 2007, the year of publication for the Schwartz’ cultural values data) for all nine ecological variables (Figs. 2–4).

Next, we explored the clustering based on Schwartz’ values and current levels of ecology, as the latter metrics are most commonly used in cross-cultural research. In the dendrogram based on Schwartz’ cultural values, many Western European countries are grouped together (blue cluster) and are distinct from the clusters which contain the Eastern European countries (purple and green,) which is broadly consistent with previous research showing regional variation in values within Europe^27^.

However, there are some clusters in the dendrogram based on Schwartz’ values that suggest cultural similarities which are not based on geographic proximity or other commonly used ways of grouping countries—such as the purple cluster, which contains Romania, Venezuela, Japan, Brazil, and the United States.

Why might this be? Examining the current levels of ecology dendrogram provides some insight into this unintuitive finding. For example, Brazil and the United States cluster closely together on the dendrograms for both Schwartz’ cultural values and their overall current social and physical ecology. Additionally, these dendrograms point to interesting avenues for future research by highlighting cases where ecological and cultural similarities diverge. For example, South Korea and Spain are in distinctly different cultural clusters but appear to have highly similar ecologies. Thus, one avenue for future research might be to investigate which factors moderate links between ecological similarity and cultural similarity.

Comparing the dendrogram built from current levels of ecology and Schwartz’ cultural values yielded a Baker’s gamma of 0.26—suggesting a moderate, positive relationship between the two. However, not all metrics of ecology show the same relationship. The Baker’s gamma comparing the Schwartz’ dendrogram to the one for MAPE (a metric of ecological unpredictability) was smaller: -0.04 (Fig. 3); for standard deviation it was larger: 0.30 (Fig. 4)—suggesting a greater degree of statistical similarity in the clustering of countries based on ecological variability and cultural values. This range of Baker’s gammas suggests that the strength of similarity between ecology and culture can vary considerably based on which metric is used to measure ecology.

## Data Availability

All calculations were conducted in R^28^ (version 4.2.0), using the *forecast*, *psych*, *foreign*, *jtools*, and *lmtest* packages^16,29–32^. The R code used to aggregate and calculate the statistical metrics of ecology is available on OSF at https://osf.io/r9msf/. Code for the dendrogram example is also available on OSF and uses the *circlize* and *dendextend* packages^33,34^.
